# Stillingia in Syphilis

**Published:** 1869-04

**Authors:** J. C. McMechan

**Affiliations:** Cincinnatti, Ohio


STILLINGIA IN SYPHILIS.—Dr. J. C. M'Mechan, Cincinnatti, Ohio, contributes the following to the *West. Jour. of Medicine:*

"In the *American Medical Recorder* for April, 1828, (vol. xiii., page 312,) Dr. Simons, recommended the use of stillingia in secondary syphilis, in place of mercury. This article, at the time attracted considerable notice, and the drug came into popular use. Doctors Lopez and Frost also wrote in favor of its use, and confirmed the views of Simons in regard to its efficiency in syphilis.

For the past few years, owing to some unaccountable reason, it has been seldom prescribed.

We have used the drug in a certain form of syphilis, and with the finest results, and have seen Dr. Dawson, Surgeon to the Cincinnati Hospital, prescribe it frequently, with the most marked effect, when other remedies had failed.

The form of syphilis in which it is most useful, is secondary, where the symptoms of tertiary are just beginning to manifest themselves, but it is also useful later in the tertiary form, in combination with iodide of potassium.

In secondary syphilis, in broken down subjects, mercury is, of course, objectionable, and if administered, cannot be carried to the point where it would have a marked effect upon the syphilis eruption. If mercury cannot be administered, there are but few remedies left to prescribe, and the principal ones, perhaps, are sarsaparilla, and iodide of potassium. The latter remedy is very good in the tertiary form, but in the secondary it has been found almost inert, having but very little, if any, effect upon the eruption. Sarsaparilla, at one time, had quite a reputation, and it was thought next to impossible for a patient to recover without its administration. It is now seldom administered, except for its moral effect, unless outside of the regular profession. Now in primary, we have iodide of mercury, (and in healthy subjects it is the proper remedy in secondary), and in tertiary, the iodide of potassium. But here is a vacancy. What is the remedy in secondary when the patient is broken down in health, or when mercury has been used without effect? There is but one remedy in the materia medica that can fill the vacancy properly, and that one is stillingia. For broken down patients with the syphilitic eruption, to patients on whom mercury has had no effect, and to patients in whom the bones have become affected and the secondary manifestations still continue, let this remedy be given."

